# Molecular mechanism of bystander effects and related abscopal/cohort effects in cancer therapy

**DOI:** 10.18632/oncotarget.24746

**Published:** 2018-04-06

**Authors:** Rong Wang, Tingyang Zhou, Wei Liu, Li Zuo

**Affiliations:** ^1^ Department of Radiation, Fifth People's Hospital of Qinghai Province, Xi Ning, Qing Hai 810007, China; ^2^ Radiologic Sciences and Respiratory Therapy Division, School of Health and Rehabilitation Sciences, The Ohio State University College of Medicine, Columbus, Ohio 43210, USA; ^3^ Interdisciplinary Biophysics Graduate Program, The Ohio State University, Columbus, Ohio 43210, USA; ^4^ Department of Radiation Oncology, Mayo Clinic Arizona, Phoenix, Arizona 85054, USA

**Keywords:** radiation therapy, non-targeted effects, p53, non-uniform irradiation, reactive oxygen species

## Abstract

Cancer cells subjected to ionizing radiation may release signals which can influence nearby non-irradiated cells, termed bystander effects. The transmission of bystander effects among cancer cells involves the activation of inflammatory cytokines, death ligands, and reactive oxygen/nitrogen species. In addition to bystander effects, two other forms of non-target effects (NTEs) have been identified in radiotherapy, as one is called cohort effects and the other is called abscopal effects. Cohort effects represent the phenomenon where irradiated cells can produce signals that reduce the survival of neighboring cells within an irradiated volume. The effects suggest the importance of cellular communication under irradiation with non-uniform dose distribution. In contrast, abscopal effects describe the NTEs that typically occur in non-irradiated cells distant from an irradiated target. These effects can be mediated primarily by immune cells such as T cells. Clinical trials have shown that application of radiation along with immunotherapy may enhance abscopal effects and improve therapeutic efficacy on non-target lesions outside an irradiated field. According to NTEs, cell viability is reduced not only by direct irradiation effects, but also due to signals emitted from nearby irradiated cells. A clinical consideration of NTEs could have a revolutionary impact on current radiotherapy via the establishment of more efficient and less toxic radiobiological models for treatment planning compared to conventional models. Thus, we will review the most updated findings about these effects and outline their mechanisms and potential applications in cancer treatment with a special focus on the brain, lung, and breast cancers.

## INTRODUCTION

Bystander effects in irradiation are defined as biological alterations manifested in un-irradiated cells when induced by signals from nearby irradiated cells within an irradiated volume [1]. Un-irradiated cells that are altered by stress signals from nearby irradiated cells are known as bystander cells. Bystander cells and irradiated cells both exhibit genetic damage, chromosome aberrations, and possibly cancer formation [2]. First discovered by Nagasawa and Little in 1992, this anomaly has been extensively researched in the past two decades and has been labeled as the radiation-induced bystander effect (RIBE) [2, 3]. Un-irradiated cells that receive bystander signals from nearby irradiated cells exhibit damaging effects like genomic instability and reduced cell survival, which are similarly observed in irradiated cells [4, 5]. The presence of the bystander effect was well described in medium transfer experiments [6, 7]. In such experiments, a cell culture medium was harvested from irradiated cells to treat un-exposed cells. Un-irradiated cells that received the radiation-conditioned medium (RCM) expressed lethal mutations and marked cell death [4]. Apart from bystander effects, there are two other classifications of signaling-mediated effects in radiation: abscopal effects and cohort effects [5]. Although these two types of effects have been associated with bystander effects by multiple sources [8–10], they could be further distinguished by Blyth *et al.,* who provided specific definitions for the three forms of such effects [5]. Abscopal effects describe the phenomenon in which irradiated tissues may emit signals to affect un-irradiated tissues outside of an irradiated volume [5, 11]. In particular, abscopal effects were observed in patients with metastatic cancers receiving radiotherapy [12]. Irradiation to a specific part of the body elicited chromosomal injury and molecular and cellular alterations in distant tissues. Following this process, increases in genetic tears, p53 involvement, DNA repair proteins, and cell death in the secluded tissues were observed [13]. These symptoms were red flags for cancer formation caused by radiation-induced abscopal effects. When one of the tumor lesions was irradiated, the non-irradiated lesions showed a significant reduction in tumor size [14]. The transmission of such effects has been suggested to be mediated by the immune system, specifically the involvement of T cells [15, 16].

Cohort effects are used to describe the interaction between irradiated cells within an irradiated volume [5], although limited research has been performed on cohort effects compared to bystander and abscopal effects. Under heterogeneous irradiation, high-dose irradiated cells may emit signals to affect low-dose irradiated cells and vice versa [5]. The identification of this effect has led to a new paradigm in radiotherapy that tissues or organs responding to ionizing radiation (IR) are affected by both the direct radiation as well as the cohort effects derived from the radiation [10]. The differences between the three IR-induced non-targeted effects are summarized in Table 1. Despite the critical roles of these three IR-mediated signaling effects in radiotherapy and cancer treatment, their underlying mechanisms and clinical implications remain elusive. This article will provide an updated summary on the molecular pathways involved in these three effects while focusing on prevalent cancers such as brain, the lungs, and breast cancer, as well as their involvement with stem cells in cancer.

**Table 1:** Summary of non-targeted effects in radiation

Non-target effects in IR	Definitions	Mechanisms	Examples
Bystander effects	IR-induced non-targeted effects in non-irradiated cells within or nearby an irradiated volume [5].	· Involve the activation of p53, ROS, NO, TGF-β1, TNF-α, PI3K, TRAIL, EGR-1, GJIC, and Fas [23, 24, 41–44, 48, 49].	· Radiation-conditioned medium transfer [4, 5] · X-ray screening [5]
Abscopal effects	IR-induced non-targeted effects in non-irradiated cells outside an irradiated volume [5].	· T cell dependent · Activation of p53, ROS, RNS, and cytokines including IL-6, IL-1α, and TNF-α, [64–67].	· IR to localized tumor [5]
Cohort effects	IR-induced non-targeted effects in irradiated cells within an irradiated volume [5].	· Potentially involve the similar mediators with bystander effects [10]	· IMRT · CT scanning [5]

Abbreviations: EGR-1, early growth response protein 1; GJIC, gap junctional intercellular communication; IL-1α, interleukin-1α; IL-6, interleukin-6; IR, ionizing radiation; NO, nitic oxide; PI3K, phosphoinositide 3-kinase; ROS, reactive oxygen species; TGF-β1, tumor growth factor-beta1; TNF-α, tumor necrosis factor-α, TRAIL, tumor necrosis factor-related apoptosis-inducing ligand.

## RADIATION-INDUCED BYSTANDER EFFECTS

### Bystander effects in brain cancer

**Figure 1:**
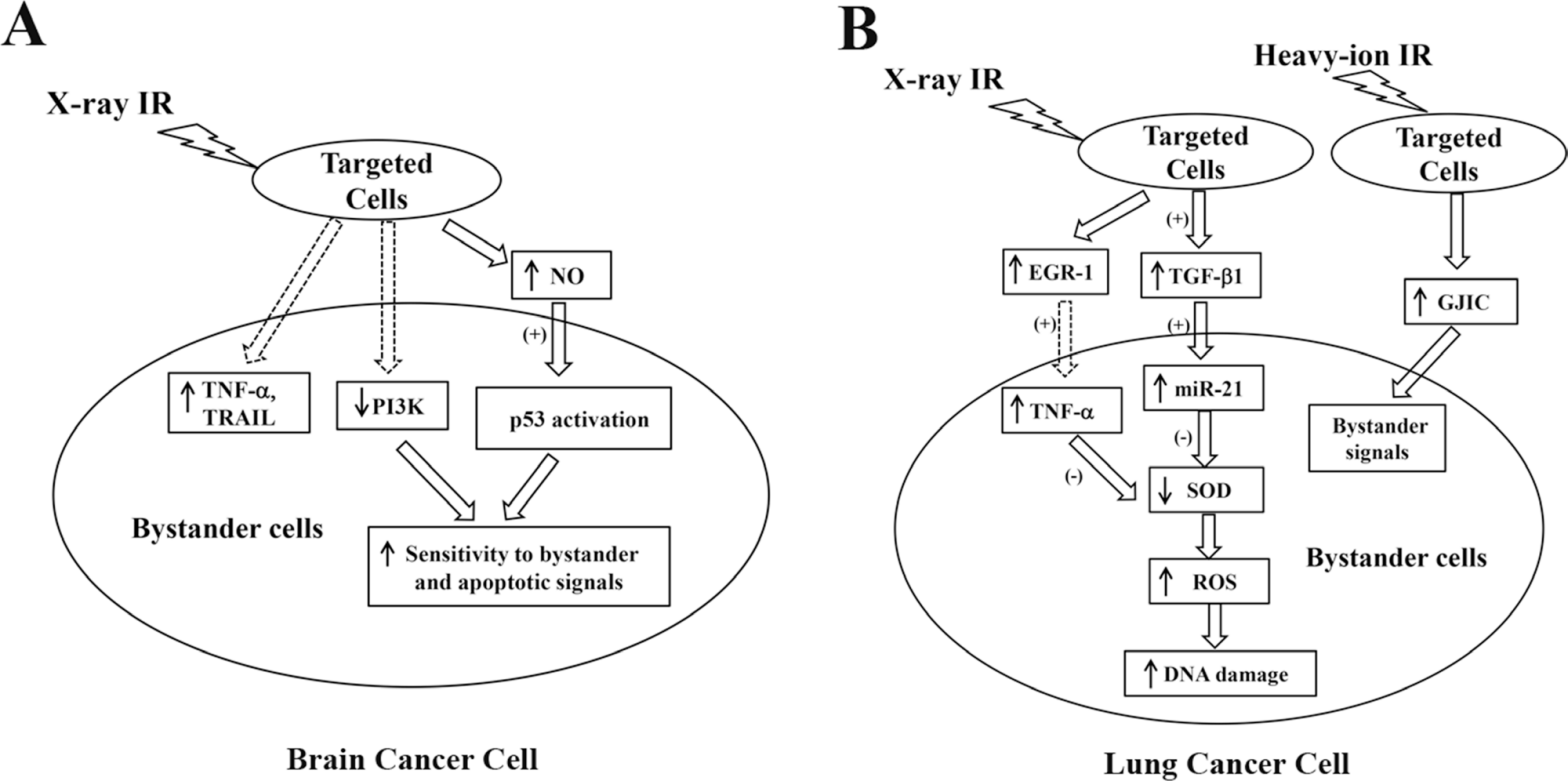
Schematic showing radiation-induced bystander signaling pathways in brain cancer cells (**A**) and lung cancer cells (**B**). EGR-1, early growth response protein-1; GJIC, gap junctional intercellular communication; NO, nitric oxide; miR-21, microRNA-21, PI3K, phosphoinositide 3-kinase; SOD, superoxide dismutase; ROS, reactive oxygen species; TNF-α, tumor necrosis factor alpha; TGF-β1, tumor growth factor-beta1; TRAIL, tumor necrosis factor-related apoptosis-inducing ligand [23, 24, 41–44, 48, 49].

The molecular mechanisms associated with bystander effects have been extensively investigated in brain cancer cell models [17–19]. For instance, bystander effects were reported to be present in glioma T98G cells when exposed to doses below 1 Gy [18]. Shao *et al.* detected significant micronuclei formation in non-irradiated cells when a small portion of the glioblastoma population was irradiated by a helium ion microbeam [19]. However, such damaging effects on non-targeted cell were abolished via the inhibition of either tumor growth factor-beta1 (TGF-β1) or inducible nitric oxide synthase (iNOS), which suggests the involvement of TGF-β1 and nitric oxide (NO) in bystander signaling cascades [19, 20]. Interestingly, the genes that activate the bystander effects also play a role in inflammation, including genes of nuclear factor of kappa B (NFκB), mitogen activated protein kinases (MAPKs), nitric oxide synthase (NOS), and cyclooxygenase 2 (COX 2) [21]. Ultimately, oxidative stress is intensified as these genes are activated, influencing inflammation and nitric oxide formation [22]. Further research on T98G cells indicated that bystander effects observed in glioblastoma could be modulated via the NO and phosphoinositide 3-kinase (PI3K) (Table 1). Specifically, bystander responses were significantly attenuated by NO inhibition but were markedly enhanced by PI3K blockers [23]. In addition, increased NO production was detected in both irradiated and non-irradiated bystander cells [23]. Since NO is hydrophobic and therefore able to pass through various membranes and cell interiors, it can readily propagate to bystander cells from cells affected by radiation without any assistance. NO can make posttranslational modifications of various regulator proteins to affect cell metabolism. Two key modifications are S-nitrosylation and tyrosine nitration, which can mediate the roles of proteins involved in NO regulation [2]. The relevant effects of NO in bystander cells are genomic instability and the accretion of DNA errors. Furthermore, previous evidence showed that NO can sensitize neuroblastomas to IR-induced apoptosis via the activation of p53 [24]. Thus, it is likely that increased NO formation activates the p53 protein in bystander cells, which renders brain cancer cells more sensitive to bystander signals (Figure 1A).

P53 is a genome guardian that induces cell cycle arrest, DNA repair, cell apoptosis, and senescence in response to stress signals [25, 26]. The p53 protein is normally kept at low levels, but is markedly increased by irradiation exposure [27, 28]. Mutations of p53 have been identified in brain, the lungs, and breast cancers [29–31]. Cancer cells bearing p53 mutations adopt stem-cell like properties, which may contribute to tumor progression and recurrence [32, 33]. It has been suggested that wild-type p53 protein is involved in the transduction of bystander signals, while alternative bystander pathways may be recruited in the absence of wild-type p53 proteins in certain cells such as keratinocytes [34]. In mutant p53 cells, NO appears to be the key mediator of bystander transduction. For instance, it was reported that p53 levels were significantly increased in glioblastoma A172 (wild-type p53) cells co-cultured with irradiated p53 mutant cells, although the p53 accumulation can be abolished by an iNOS inhibitor [35]. Significant accumulation of p53 was observed in bystander cells, which may account for bystander-induced cell death via induction of apoptosis [36]. Furthermore, multiple lines of evidence have indicated that p53 may act to regulate bystander effects involving both intra- and extracellular ROS formation [27, 36, 37]. Specifically, NAD(P)H oxidase (Nox)-induced ROS play a key role in stimulating p53 expression in bystander cells [34]. This is supported by the evidence that superoxide dismutase (SOD) treatment effectively diminishes the radiation-induced p53 accumulation in bystander cells [36]. Upregulation of p53 has been shown to induce intracellular ROS production, which in turn initiates apoptotic cascades [27, 38]. Therefore, it is likely that irradiated p53 wild-type cells transmit bystander signals through ROS-involved pathways, while irradiated cells with p53 mutation release NO-dependent bystander signals [37]. In addition, PI3K activation has been shown to play an important role in cancer cell survival [23]. PI3K can trigger AKT signaling, which markedly increased the cell sensitivity to tumor necrosis factor (TNF)-induced apoptosis [39]. The inhibition of PI3K survival cascades has been shown to sensitize tumor cells to cytokines including TNF-α and tumor necrosis factor-related apoptosis-inducing ligand (TRAIL) [23]. Since both TNF-α and TRAIL are potential signaling molecules involved in bystander effects, the blocking of the PI3K pathway may predispose the tumor cells to be more sensitive to bystander effects (Figure 1A) [23].

### Bystander effects in lung cancer

Significant bystander effects have been reported in lung cancer cells under different treatment conditions [40–42]. For example, Jiang *et al.* detected elevated oxidative stress and DNA damage in H1299 non-small-cell lung cancer cells that received a RCM. H1299 cells were irradiated with 5 Gy of X-ray. After either 1 h or 18 h from IR, the RCM was collected and transferred to un-irradiated H1299 cells [43]. Interestingly, the 1 h-RCM and 18 h-RCM induced different cellular responses in bystander cells (the un-irradiated cells that received the RCM). The 1 h-RCM caused elevation of both intracellular ROS and DNA damage in bystander cells, which was not observed in 18 h-RCM-treated cells. However, the 18 h-RCM markedly decreased bystander cell proliferation, and 1 h-RCM did not [43]. These effects can be abolished by TGF-β1 inhibition in both irradiated and bystander cells. In addition, microRNA (miR)-21 expression was found to be upregulated in bystander cells when treated with 1h-RCM, but downregulated in cells receiving 18 h-RCM. TGF-β1 inhibition can reduce such miR-21 dysregulation in bystander cells. These results collectively delineate a bystander cascade involving TGF-β1/miR-21/ROS (Figure 1B and Table 1) [43]. Furthermore, the relationship between miR-21 and ROS has been well established in 293 FT cells [44]. It was observed that miR-21 overexpression is associated with decreased SOD 2 and 3 levels [44]. Since SOD is an important scavenger for superoxide (O_2_^·–^, a major type of ROS), the downregulation of SOD 2 and SOD 3 by miR-21 represents a possible mechanism for miR-21-induced ROS accumulation in 1 h-RCM bystander cells (Figure 1B) [43–45]. Through the generation of superoxide and free radicals, mitochondria can exacerbate injury to cells induced by bystander effects [46, 47].

The involvement of TNF-α in the bystander signaling pathway was also evidenced in a study by Shareef *et al.,* which investigated the effects of X-ray irradiation in the lung cancer cell line A549 (Table 1) [41]. Enhanced TNF-α release and decreased cell viability was observed in un-irradiated A549 cells after RCM exposure, while the neutralization of TNF-α improved bystander cell survival [41]. The activation of TNF-α potentially contributes to increased ROS generation in response to bystander signals via SOD inhibition (Figure 1B) [44, 48]. It was further proposed by Shareef *et al.* that IR-induced early growth response protein (EGR)-1 may be responsible for the upregulation of TNF-α that was observed in bystander cells (Figure 1B) [41]. Apart from soluble factors within a cell culture medium, gap junctional intercellular communication (GJIC) has been suggested as a key mechanism for heavy ion-induced bystander effects in physically contacting cells (Figure 1B and Table 1) [42, 49]. For example, in a study by Harada *et al*., 1.3 × 10^6^ ± 0.4 × 10^6^ of A549 cells were cultured in a confluent condition to ensure physical contact. A small fraction of cells (0.0001–0.002%) in the dish were irradiated by carbon-ions using a microbeam (18.3 MeV/u). The irradiation exaggerated cell death by 8–14%, indicating the presence of bystander effects [42]. Such responses were abolished by GJIC inhibition and enhanced with GJIC stimulators. Furthermore, the heavy ion-induced bystander phenomenon was not observed when cells were cultured at low concentrations with a lack of physical contact. These observations together suggest that bystander effects induced by heavy ion-irradiation (e.g., carbon ions) depend on GJIC [42].

The transmission pattern of bystander effects has been well described in a normal lung fibroblast model [40]. V79 cells (lung fibroblast cell line) were irradiated by low doses of X-ray radiation (0–2 Gy) [40]. When a single cell was irradiated by a microbeam, 10% of the cell death was detected in the culture dish. Such non-targeted cell killing effects were dose-dependent below 0.2 Gy but were saturated above 0.2 Gy, suggesting high sensitivity to bystander effects in such cell lines [40]. Furthermore, an examination of the distribution of damaged cells indicated that cell death spread uniformly over the culture dish, regardless of the distance from the irradiated cell (up to 3 mm). However, a significant clustering effect of damaged cells was observed [40]. These observations indicate that a chain reaction was induced during the transmission of bystander effects (i.e., un-irradiated cells that receive signals from irradiated cells can themselves release bystander signals to decrease the survival of neighboring cells) [40].

### Bystander effects in breast cancer

Radiation-induced bystander effects were also reported in breast cancer cells but with relatively low dose sensitivity [50]. Specifically, breast carcinoma cells may only show bystander responses after IR sensitization [50]. In the study by Luce *et al*., T-47D breast cancer cells received a 10-Gy γ-irradiation. Six days after IR, the RCM was transferred to non-irradiated cells [50]. Control cells received a non-radiated conditioned medium (NRCM) and cell death was evaluated 24 h after the medium transfer. The study found that RCM did not lead to more cell death compared to the control. However, if the breast carcinoma cells were sensitized by 10 Gy of irradiation 24 h before the medium transfer, the cells receiving RCM showed a significantly higher cell death rate as compared to NRCM-treated cells [50]. These results indicate that breast cancer cells are more resistant to bystander effects compared to brain and lung cancer cells. However, IR exposure can substantially increase the sensitivity of breast carcinoma to bystander signals [50]. Furthermore, Fas, TNF-α, and TRAIL were all proposed as potential signaling molecules involved in bystander activation in the study (Table 1) [50]. Specifically, enhanced expression of death receptors for Fas, TNF-α, and TRAIL were all observed after γ-irradiation in breast cancer cells. This was followed by increased formation of Fas ligands, TNF-α, and TRAIL, contributing to delayed cell apoptosis after IR exposure [50]. It was further reported that soluble forms of these three ligands were released from irradiated breast cancer cells, which may account for bystander transduction to neighboring un-irradiated cells [50].

### Bystander effects in stem cells

**Figure 2:**
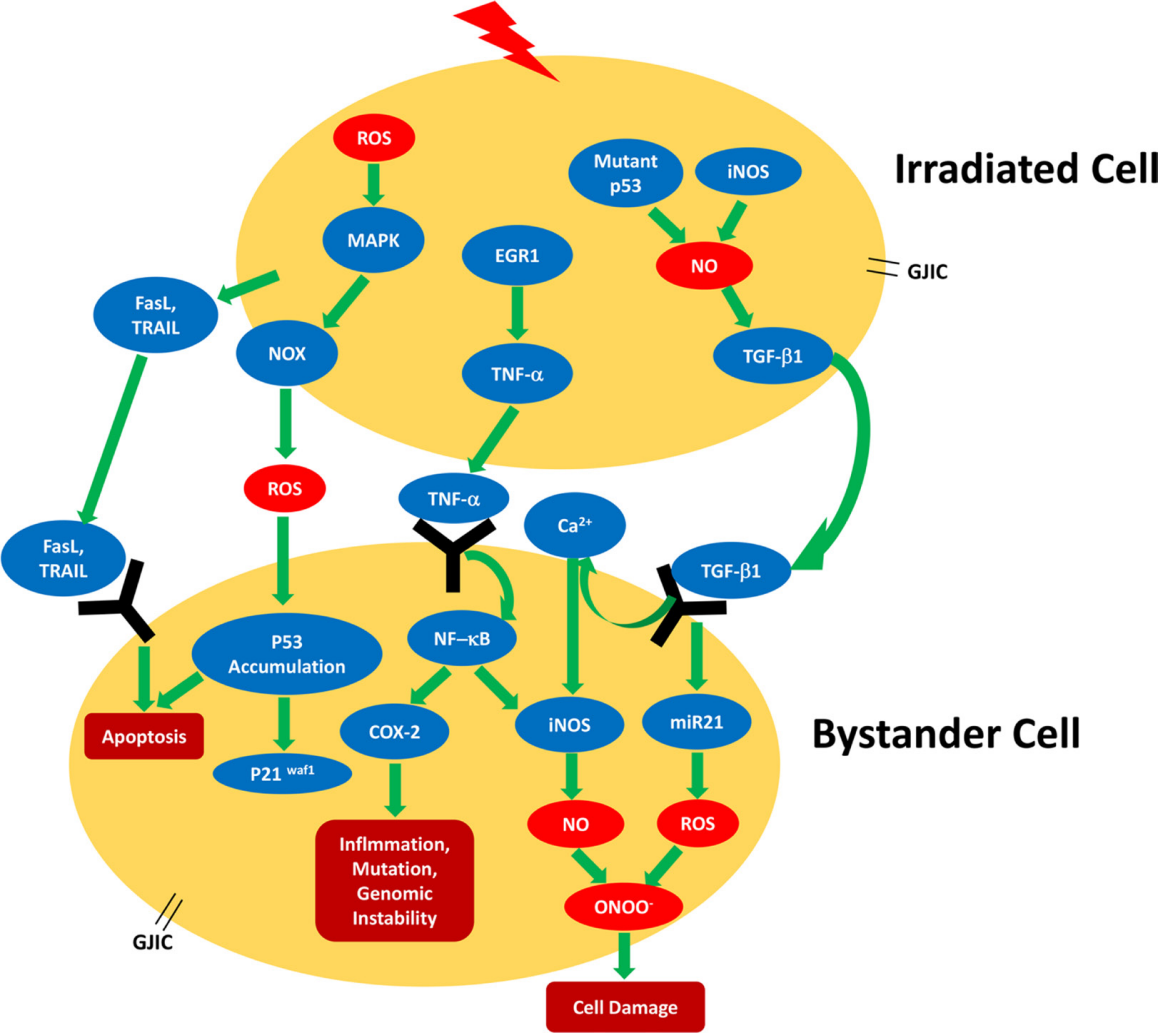
Schematic summarizing the radiation-induced bystander signaling pathways found in different cell types Lightning bolt indicates irradiation; COX-2, cyclooxygenase 2; EGR-1, early growth response protein-1; FasL, Fas ligand; GJIC, gap junctional intercellular communication; iNOS, inducible nitric oxide synthase; NO, nitric oxide; NOS, nitric oxide synthase; NOX, NAD(P)H oxidase; miR-21, microRNA-21; ROS, reactive oxygen species; TNF-α, tumor necrosis factor alpha; TGF-β1, tumor growth factor-beta1; TRAIL, tumor necrosis factor-related apoptosis-inducing ligand [41–43, 47, 49, 50, 76, 81–85].

Many solid tumors such as breast cancer and glioblastoma bear a small population of cancer stem cells within the tumor with extensive self-renewal capability, which may account for cancer metastasis as well as radiation resistance [51, 52]. The radio-resistance of cancer stem cells has been attributed to their enhanced antioxidant defense, lower ROS levels, activation of DNA damage checkpoints, and more effective DNA repair mechanisms [53]. Therefore, it is of great interest to explore whether these features may render stem cells more resistant to bystander signals than normal cancer cells. Research has shown that bystander effects can be induced in murine hematopoietic stem cells (HSC) but not in human bone-marrow mesenchymal stem cells (hMSC) [54–56]. For example, decreased clonogenic survival and genomic instability were observed in murine stem cells after the cells were co-cultured with or received medium from irradiated stem cells [57]. Similar results were produced by Lorimore *et al.* In their investigation, both CBA/Ca mice (myeloid leukemia susceptible strain) and C57BL/6 mice received γ irradiation of 4 Gy, a dose sufficient to induce myeloid leukemia in CBA/Ca mice. Bone marrow cell medium derived from the irradiated mice was transferred to normal murine HSC and chromosomal stability was analyzed in bystander cells [56]. The results showed that bystander-related genomic instability can be induced only when both the medium-donor cells and medium-receiver cells were derived from CBA/Ca strain. Previous studies have shown that macrophages in the hematopoietic system respond differently between CBA/Ca and C57BL/6 strains to a leukemogenic dose of irradiation [56]. The macrophages from CBA/Ca mice demonstrated increased pro-inflammatory phenotypes, while C57BL/6 macrophages exhibited enhanced anti-inflammatory phenotypes when exposed to irradiation [56]. Therefore, it was speculated that only macrophages from the CBA/Ca strain can be activated by irradiation to release bystander signals and induce cytogenetic aberrations in bystander cells [56]. This was supported by the observation that the cell culture medium conditioned by macrophages from irradiated CBA/Ca strain led to chromosomal instability in medium-receiver cells [56]. In addition, TNF-α, ROS, and NO have been suggested as critical signaling molecules required to activate bystander pathways in HSC, as treatment with their respective inhibitors significantly attenuated genomic instability in bystander cells (Table 1) [56]. However, contradictory results were provided by Sokolove *et al.* who observed no bystander-associated DNA damage or apoptosis in hMSC that received RCM [54]. Currently, it remains unclear why radiation-induced bystander effects are evident in some cell lines but virtually absent in other cells, and this may require further investigation [54]. The bystander mechanisms that underlie different cancer cells are summarized in Figure 2.

## RADIATION-INDUCED ABSCOPAL EFFECTS

In the treatment of cancer, radiation therapy could have far-reaching or delayed influence on cells, even though it has been generally accepted that local modalities do not have systemic consequences [34]. Unlike the bystander effect, which pertains to cells adjacent to irradiated cells, the abscopal effect is much further-reaching [58]. Researchers also have a better grasp on the bystander effect and related mechanisms, while the abscopal effect relies on clinical changes due to radiation [58]. These clinical changes occasionally arise distant from the radiation site, and it is likely that they are the result of factors released from irradiated cancer cells as well as correlated immune cells [59]. Abscopal effects have also been found to hold great potential in radiotherapy. Golden *et al.* reported that abscopal effects can be detected in patients receiving a combined treatment of radiotherapy and immunotherapy [60]. In their study, 41 patients with metastatic cancer were injected with granulocyte-macrophage colony-stimulating factor (GM-CSF, a type of immune-based therapy) and received fractionated IR on one of the tumor lesions. After 7–8 weeks, a significant abscopal response (> 30% tumor shrinking of untreated lesions) was detected in 27% of the patients, including two best-represented groups of breast cancer and non-small-cell lung cancer [60]. Therefore, it is important to use fractionated radiation together with immune interventions to initiate abscopal effects. The lack of marked abscopal responses in the other patients is potentially due to immunosuppression in specific tumor context [14]. This research represents a novel cancer treatment strategy that combines radiation therapy and immunotherapy to control tumor expression via abscopal-mediated pathways. One instance of the abscopal effect, reported by Postow *et al.*, involved the treatment of a melanoma patient with ipilimumab and radiation therapy. Ipilimumab is a medicine that can be used enhance the immune response to NY-ESO-1, an antigen existing in 30–40% of advanced melanoma population. When Ipilimumab was administered in combination with conventional radiotherapy, a systematic response in non-irradiated tissue was clearly observed including regressed lesions in both hilar lymph node and the spleen [61]. Another case of the abscopal effect, documented by Okuma *et al.,* involved the pulmonary invasion of hepatocellular carcinoma, suggesting the broad spectrum of cancers influenced by the abscopal effect [62]. Abscopal effects have been shown in a patient with uterine cervical carcinoma exhibiting lymph node metastasis. By directly irradiating the whole pelvis, the pelvic lesion was completely eliminated, and para-aortic lymph node metastases were also eradicated [63].

As mentioned previously, abscopal effects refer to the anti-tumor effects on lesions distant to the irradiated site in which the immune system may play a key role [14, 64]. Specifically, radiation has been suggested to induce local inflammation and augment T-cell activation, leading to cancer cell elimination via T-cell-dependent pathways (Table 1). This theory may explain why abscopal effects are more frequently observed in individuals with strong immune systems [65]. Furthermore, in response to radiotherapy, released cytokines have been shown to play critical roles in abscopal responses. The suppression of tumors due to the abscopal effect is likely regulated by a systemic antitumor effect caused by the discharge of cytokines into the bloodstream [65]. For example, Khan *et al.* reported that when irradiation was delivered to a part of the lungs in rats, the genomic damage was observed in non-irradiated regions of the lung as well [66]. However, pre-treatment with Cu-Zn SOD or NO inhibitor attenuated such damaging effects in non-targeted areas [66]. Additionally, cytokines including interleukin (IL)-6, IL-1α, and TNF-α were also significantly elevated after irradiation, which was accompanied by macrophage activations [67]. Together these results suggest the involvement of cytokines, ROS, and NO in the activation of abscopal effects (Table 1) [65]. Furthermore, the research by Camphausen *et al.* examined the roles of p53 in mediating abscopal effects in mice [64]. Both wild-type p53 mice (C57BL/6) and p53-null mice (B6.129S2-*Trp53^tm1Tyj^*) received irradiation on their legs with five fractions of either 10-Gy or 2-Gy. Both Lewis lung carcinoma (LLC) and fibrosarcoma (T241), were implanted to a distant location from irradiation sites. The research showed that leg irradiation markedly reduced the growth rate of both LLC and T241 tumors in C57BL/6 mice compared to non-irradiated animals. This result suggests that abscopal effects are not tumor specific. Furthermore, tumor growth was not affected by leg irradiation in p53-null mice, which indicates that p53 could be an important mediator in eliciting such effects [64]. Additionally, IR with 10-Gy fractions has been shown to induce a more prominent tumor inhibition effect than 2-Gy fractions, confirming the dose-dependence of abscopal effects [64].

It has been found that X-irradiation elicits anti-inflammatory properties at low levels (1 < Gy) and prompts anti-tumor immune effects at high levels. Evidence suggests that these effects are mediated by direct DNA damage along with non-targeted mechanisms such as bystander and abscopal effects and genomic instability. Preclinical studies imply that there are still debates on optimal radiation doses and fractionation schemes for eliciting systemic anti-tumor (abscopal) effects to eradicate cancer cells, especially when used in combination with immune therapies [68]. More experiments should be conducted in order to determine the optimal combination and order of different treatment modalities including radiotherapy, chemotherapy, and immune therapy for systemic and specific tumor-suppression responses in animal models [68]. Immunotherapy compounds that imitate, enhance, or recruit the involvement of the host immune system for optimal treatment effects are known as immune response modifiers (IRM) [69]. Although the involvement of host T cells in tumor response to irradiation were identified for more than thirty years, the associated mechanisms have only just surfaced in the past ten years [69]. The increasing use of radiation as an immunological ancillary component has been shown as the potential for developing new combinations of both radiotherapy and immunotherapy. Evidence proposing that radiation may work as an IRM, would advocate for a fundamental change in how radiation is used in cancer treatment [69].

Although it is generally accepted that abscopal effects are elicited by T cell activation, it remains difficult to predict the occurrence of abscopal effects in patients receiving radiotherapy [70]. According to abscopal effects, IR may induce cancer cells to secrete pro-inflammation cytokines, which then recruit T cells to achieve systematic anti-tumor effects [71, 72]. However, this is not highly likely because regulation of abscopal effects relies on a delicate balance between immune suppression and immune activation [72]. Unfortunately, IR alone is seldom successful in pushing the balance towards the immune activation, as evidenced by the rare cases of abscopal effects in clinical settings [70]. However, with the assistance of immune boosting therapy, the occurrence of abscopal effects can be markedly improved, as observed among one third of patients in previous research [70]. Therefore, the immune environment of specific tumors such as the availability of local dendritic cells and patients’ immunity could be important determinants of abscopal effects [70]. Furthermore, it has been suggested that sizes of irradiated tumor may influence abscopal effects. Larger tumor tissues may be able to release more antigens in response to irradiation that potentially intensify abscopal effects than that from smaller size. However, a larger tumor could also be characterized by more hypoxic areas, which are known to be immunosuppressive [70]. Therefore, it is critical to take these factors into consideration when predicting or using immune therapy to augment abscopal effects. Further studies are mecessary to explore the effects of IR on immune mircro-environment to develop reliable predictors of abscopal effects in clinical practice.

## RADIATION-INDUCED COHORT EFFECTS

According to cohort effects, the net response of a cell to IR includes both direct radiation effects and cellular responses to cohort signals emitted from the neighboring irradiated cells. The contributions of the direct radiation and cohort effects may be dependent on the dose and quality of irradiation [5]. Compared to the other two scenarios, radiation-elicited cohort effects are less studied and are usually referred to as “bystander effects” without distinction in many studies [9, 10, 21]. However, there are several differences between bystander and cohort effects. First, cohort effects are typically observed in irradiated cells while bystander effects were mostly found in non-irradiated cells [5]. Second, they have different saturated doses. Bystander effects have been described as a low-dose phenomenon because they can be easily saturated with relatively lower doses, typically ranging from levels of mGy to cGy [73–77]. For example, in a study with human fibroblasts, non-irradiated cells (i.e., bystander cells) were cultured in the cell media obtained from X-ray irradiated cells. It was found that bystander cells showed reduced survivals when the irradiated cells were subjected to a dose from 0-0.5 Gy. However, the survival of bystander cells did not change when the irradiation dose were higher than 0.5 Gy, suggesting that bystander effects were saturated at 0.5 Gy in these cells [76]. In contrast, cohort effects have been frequently observed in non-uniform irradiation with relatively higher irradiation doses ranging from 1–8 Gy in various cell lines [9, 10].

Cohort effects may play a significant role in heterogeneous irradiation such as intensity modulated radiotherapy (IMRT) because high-dose irradiated cells may emit signals to affect the nearby low-dose irradiated cells, and vice versa, thus potentially enhancing the overall cell-killing efficiency of heterogeneous irradiation at low-dose regions [5, 9]. This was supported by the evidence that under gradient irradiation (GI), cancer cell survival rate was largely reduced at low-dose irradiated regions but was improved at high-dose irradiated regions in comparison to that under similar doses with uniform irradiation (UI) [9]. In other words, the cellular response to non-uniform irradiation seemed to “average out” across the entire dish culture via cohort effects [9]. Our previous study applied such effects in radiotherapy and proposed a dosing scheme of GI superior to conventional UI [10]. Breast cancer cells (MCF-7) cultured on 3-cm diameter dishes received either GI (8-2 Gy from the center to edge) or UI (uniformly 5 Gy). Under GI, the center of the dish received the highest doses, which was modulated to be gradually decreased to 2 Gy at the edge of the dish. Cell death and oxidative stress were evaluated following IR treatment. It was found that GI induced more cell death and ROS formation than UI at different dose regions at 48h after IR [10]. These results suggest that the high-dose irradiated cells may emit cohort signals to increase the oxidative stress and reduce cell survival in low-dose regions. These findings indicate the advantages of using GI over UI in cancer treatment due to the attenuated radiation scattering effects of GI on nearby healthy tissues outside the irradiation field [10]. Although the molecular mechanisms underlying cohort effects remain largely unknown, it is speculated that the signaling molecules involved in bystander effects such as TGF-β1, ROS, and NO may play critical roles [10, 43]. More investigations are warranted to understand the redox pathways and transmission pattern of cohort effects with the aim of developing safer and more effective cancer therapies.

## CONCLUSIONS AND PERSPECTIVES

Conventional paradigm of radiotherapy focuses on the direct IR effects initiated within the targeted cells. Cell death has been shown to be the result of high amounts of irradiation energy infiltrating DNA structures within the nucleus, primarily by inducing double-strand breaks. However, this paradigm is not devoid of doubt. A reevaluation of the present model of cell killing by radiation has been prompted by recent developments in the understanding of bystander mechanisms as well as the emerging technology which allows for single cell targeting with microbeams. Current research implies that DNA damage does not directly elicit the cell death response. Instead, genetic repair may play a central role in modulating downstream consequences in cells affected by bystander effects [78–80]. Accumulating studies have suggested the importance of IR-induced non-targeted effects in cancer treatment including bystander effects, abscopal effects, and cohort effects. They play important roles in mediating cell survival in non- or less-irradiated cells via the communication with irradiated cells. The molecular mechanisms underlying these effects remain to be elucidated but may involve the activation of ROS, NO, cytokines, and the immune system. A further exploration of the transmission pattern and the underlying mechanisms of these effects are needed to maximize cancer-killing efficiency and decrease adverse treatment effects during radiation.

## Abbreviations

COX 2: cyclooxygenase 2
FasL: Fas ligand
GI: gradient irradiation
GJIC: gap junctional intercellular communication
GM-CSF: granulocyte-macrophage colony-stimulating factor
hMSC: human bone-marrow mesenchymal stem cells
HSC: hematopoietic stem cells
IL: interleukin
IMRT: intensity modulated radiotherapy
iNOS: inducible nitric oxide synthase
IR: ionizing radiation
IRM: immune response modifiers
LLC: Lewis lung carcinoma
MAPKs: mitogen activated protein kinases
miR: microRNA
NFκB: nuclear factor of kappa B
NO: nitric oxide
NOS: nitric oxide synthase
Nox: NAD(P)H oxidase
NRCM: non-radiated conditioned medium
NTE: non-targeted effects
PI3K: phosphoinositide 3-kinase
RCM: radiation-conditioned medium
RIBE: radiation-induced bystander effects
ROS: reactive oxygen species
SOD: superoxide dismutase
TGF-β1: tumor growth factor-beta1
TNF-α: tumor necrosis factor alpha
TRAIL: tumor necrosis factor-related apoptosis-inducing ligand
UI: uniform irradiation.
